# Data of RNA sequencing of pearl millet panicles treated with a high temperature

**DOI:** 10.1016/j.dib.2024.110074

**Published:** 2024-01-18

**Authors:** Xichao Lou, Shashi Kumar Gupta, Tetsuo Takano, Daisuke Tsugama

**Affiliations:** a Asian Research Center for Bioresource and Environmental Sciences (ARC-BRES), Graduate School of Agricultural and Life Sciences, The University of Tokyo, 1-1-1 Midori-cho, Nishi-tokyo-shi, Tokyo 188-0002, Japan; b International Crops Research Institute for Semi-Arid Tropics (ICRISAT), Patancheru, Telangana 502 324, India

**Keywords:** Pearl millet, RNA sequencing, Transcriptome, High temperature, Panicle, Seed

## Abstract

Pearl millet (*Pennisetum glaucum*) is a cereal crop that can grow and set seeds even under drought, high temperatures and nutrient-poor conditions. Panicles of two pearl millet cultivars that differ in seed-setting rates were exposed to two different high-temperature treatments at three different developmental stages with three replicates, and RNA was prepared from these panicles. The resulting RNA samples were subjected to sequencing with the Illumina NovaSeq 6000 sequencer. The obtained data were 150-base-paired-end reads and were approximately 5 Gb/sample in total. These read data were deposited as those for a project in the NCBI (National Center for Biotechnology Information) BioProject database.

Specifications Table

**Table:** 

Subject	Plant Science
Specific subject area	RNA present in panicles of pearl millet (*Pennisetum glaucum*) exposed to a high temperature
Data format	Raw Analyzed
Type of data	Table, graph, figure Figure
Data collection	ICMB00333 is a pearl millet cultivar with a high seed-setting rate under a high temperature, and ICMB00555 is a cultivar with a low seed-setting rate under a high temperature. ICMB00333 and ICMB00555 plants were grown in a growth chamber under a 28°C day/20°C night condition until they reached the booting, panicle head-emerging or anther-emerging stage. Their panicles were then exposed to 42°C in either the growth chamber or water. RNA samples were prepared from florets in these panicles and subjected to RNA sequencing (RNA-Seq) with the Illumina NovaSeq 6000 sequencer.
Data source location	Tanashi Campus of The University of Tokyo Nishi-tokyo-shi, Tokyo, Japan North latitude 35°74’ and east longitude 139°54’
Data accessibility	Repository name: NCBI (National Center for Biotechnology Information) BioProject (for RNA-Seq-derived reads); figshare (for tables and graphs) Data identification number: PRJNA926343 (for RNA-Seq-derived reads); 10.6084/m9.figshare.24532792 (DOI, for tables and graphs) Direct URL to data: https://www.ncbi.nlm.nih.gov/bioproject/PRJNA926343 (for RNA-Seq-derived reads) https://doi.org/10.6084/m9.figshare.24532792 (for tables and graphs)

## 1 Value of The Data

• These data can be used to obtain expression levels of genes in pearl millet panicles exposed to a high temperature
• These data can also help narrow down genes that determine differences in seed-setting rates under high temperatures between pearl millet cultivars
• Pearl millet researchers and breeders will benefit from these data
• Researchers and breeders for other crops can also benefit from these data.

## 2 Background

Pearl millet (*Pennisetum glaucum*) is in general tolerant to a high temperature as well as to drought and nutrient-poor conditions. Nevertheless, seed-setting rates of pearl millet can be decreased by 40°C or a higher temperature, and its extent differs between cultivars. For example, the cultivar ICMB00333 can maintain the seed-setting rate under a high temperature, whereas ICMB00555 cannot. Such a high temperature can be reached during the seed-setting stage in regions where pearl millet is cultivated. It is therefore relevant to pearl millet production to identify or develop cultivars that can maintain its seed-setting rate under a high temperature and to understand mechanisms underlying such high temperature tolerance [1]. In pearl millet, only pistils are visible at the booting and panicle emergence stages, and the anther emergence stage follows them. The high temperature-dependent decrease of seed-setting rates of pearl millet is more severe when a high temperature is imposed at the booting or panicle emergence stage than when it is imposed at a later stage. This suggests that the pistils at early developmental stages are more sensitive to a high temperature than those at later stages and pollen grains [2].

## 3 Data Description

**Figure 1:**
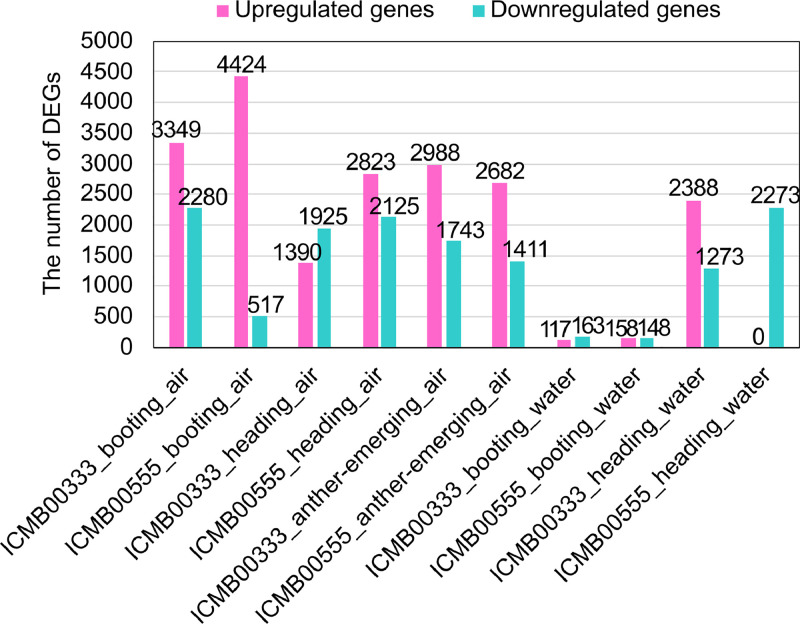
The numbers of the DEGs detected. “booting”, “heading” and “anther-emerging” correspond to the booting, panicle emergence and anther emergence stages, respectively, when the panicles were treated with 42°C. For “air”, a growth chamber was used to impose 42°C, and for “water”, a water bath was used. The DEGs were obtained on the basis of TPM as the normalized expression level for each gene as described in “EXPERIMENTAL DESIGN, MATERIALS AND METHODS”. The exact numbers of the DEGs are presented in the figure.

Seed-setting rates of ICMB00333 and ICMB00555 plants were assessed after they were grown and exposed to 42°C under a controlled condition in the laboratory. The resulting data were deposited in the figshare repository (https://doi.org/10.6084/m9.figshare.24532792). Panicles of these cultivars were exposed to 42°C water or air at the booting, panicle emergence or anther emergence stage, and subjected to RNA sequencing (RNA-Seq). The resulting reads were deposited in the NCBI Sequence Read Archive (SRA) database (https://www.ncbi.nlm.nih.gov/sra) and are available with the NCBI BioProject accession number PRJNA926343. These are summarized in Table 1. The reads were mapped to the pearl millet reference genome [3]. Resulting read counts and transcripts per million (TPM) for each gene (or transcript) and a list of differentially expressed genes (DEGs) (i.e., genes either upregulated or downregulated by the 42°C treatment) as well as the seed-setting rates of ICMB00333 and ICMB00555 were deposited in figshare (https://doi.org/10.6084/m9.figshare.24532792, Table 2). The numbers of DEGs are presented in Fig. 1.

**Table 1:** Samples and the SRA accession numbers associated with PRJNA926343.

BioSample	SRA	ID	Cultivar	Stage	℃	Treated_in	Replicate
SAMN32868483	SRR23185799	AS1325B	ICMB00333	booting	28	growth chamber	1
SAMN32868471	SRR23185812	AS2325B	ICMB00333	booting	28	growth chamber	2
SAMN32868459	SRR23185826	AS3325B	ICMB00333	booting	28	growth chamber	3
SAMN32868480	SRR23185802	AS1342B	ICMB00333	booting	42	growth chamber	1
SAMN32868468	SRR23185816	AS2342B	ICMB00333	booting	42	growth chamber	2
SAMN32868456	SRR23185828	AS3342B	ICMB00333	booting	42	growth chamber	3
SAMN32868477	SRR23185806	AS1525B	ICMB00555	booting	28	growth chamber	1
SAMN32868465	SRR23185819	AS2525B	ICMB00555	booting	28	growth chamber	2
SAMN32868453	SRR23185792	AS3525B	ICMB00555	booting	28	growth chamber	3
SAMN32868474	SRR23185809	AS1542B	ICMB00555	booting	42	growth chamber	1
SAMN32868462	SRR23185822	AS2542B	ICMB00555	booting	42	growth chamber	2
SAMN32868450	SRR23185842	AS3542B	ICMB00555	booting	42	growth chamber	3
SAMN32868482	SRR23185800	AS1325H	ICMB00333	panicle emergence	28	growth chamber	1
SAMN32868470	SRR23185813	AS2325H	ICMB00333	panicle emergence	28	growth chamber	2
SAMN32868458	SRR23185825	AS3325H	ICMB00333	panicle emergence	28	growth chamber	3
SAMN32868479	SRR23185803	AS1342H	ICMB00333	panicle emergence	42	growth chamber	1
SAMN32868467	SRR23185817	AS2342H	ICMB00333	panicle emergence	42	growth chamber	2
SAMN32868455	SRR23185829	AS3342H	ICMB00333	panicle emergence	42	growth chamber	3
SAMN32868476	SRR23185807	AS1525H	ICMB00555	panicle emergence	28	growth chamber	1
SAMN32868464	SRR23185820	AS2525H	ICMB00555	panicle emergence	28	growth chamber	2
SAMN32868452	SRR23185804	AS3525H	ICMB00555	panicle emergence	28	growth chamber	3
SAMN32868473	SRR23185810	AS1542H	ICMB00555	panicle emergence	42	growth chamber	1
SAMN32868461	SRR23185823	AS2542H	ICMB00555	panicle emergence	42	growth chamber	2
SAMN32868449	SRR23185843	AS3542H	ICMB00555	panicle emergence	42	growth chamber	3
SAMN32868484	SRR23185798	AS1325A	ICMB00333	anther emergence	28	growth chamber	1
SAMN32868472	SRR23185811	AS2325A	ICMB00333	anther emergence	28	growth chamber	2
SAMN32868460	SRR23185824	AS3325A	ICMB00333	anther emergence	28	growth chamber	3
SAMN32868481	SRR23185801	AS1342A	ICMB00333	anther emergence	42	growth chamber	1
SAMN32868469	SRR23185814	AS2342A	ICMB00333	anther emergence	42	growth chamber	2
SAMN32868457	SRR23185827	AS3342A	ICMB00333	anther emergence	42	growth chamber	3
SAMN32868478	SRR23185805	AS1525A	ICMB00555	anther emergence	28	growth chamber	1
SAMN32868466	SRR23185818	AS2525A	ICMB00555	anther emergence	28	growth chamber	2
SAMN32868454	SRR23185785	AS3525A	ICMB00555	anther emergence	28	growth chamber	3
SAMN32868475	SRR23185808	AS1542A	ICMB00555	anther emergence	42	growth chamber	1
SAMN32868463	SRR23185821	AS2542A	ICMB00555	anther emergence	42	growth chamber	2
SAMN32868451	SRR23185815	AS3542A	ICMB00555	anther emergence	42	growth chamber	3
SAMN32868508	SRR23185830	WS1325B	ICMB00333	booting	28	water bath	1
SAMN32868500	SRR23185838	WS2325B	ICMB00333	booting	28	water bath	2
SAMN32868492	SRR23185789	WS3325B	ICMB00333	booting	28	water bath	3
SAMN32868506	SRR23185832	WS1342B	ICMB00333	booting	42	water bath	1
SAMN32868498	SRR23185840	WS2342B	ICMB00333	booting	42	water bath	2
SAMN32868490	SRR23185790	WS3342B	ICMB00333	booting	42	water bath	3
SAMN32868504	SRR23185834	WS1525B	ICMB00555	booting	28	water bath	1
SAMN32868496	SRR23185784	WS2525B	ICMB00555	booting	28	water bath	2
SAMN32868488	SRR23185794	WS3525B	ICMB00555	booting	28	water bath	3
SAMN32868502	SRR23185836	WS1542B	ICMB00555	booting	42	water bath	1
SAMN32868494	SRR23185787	WS2542B	ICMB00555	booting	42	water bath	2
SAMN32868486	SRR23185796	WS3542B	ICMB00555	booting	42	water bath	3
SAMN32868507	SRR23185831	WS1325H	ICMB00333	panicle emergence	28	water bath	1
SAMN32868499	SRR23185839	WS2325H	ICMB00333	panicle emergence	28	water bath	2
SAMN32868491	SRR23185793	WS3325H	ICMB00333	panicle emergence	28	water bath	3
SAMN32868505	SRR23185833	WS1342H	ICMB00333	panicle emergence	42	water bath	1
SAMN32868497	SRR23185841	WS2342H	ICMB00333	panicle emergence	42	water bath	2
SAMN32868489	SRR23185791	WS3342H	ICMB00333	panicle emergence	42	water bath	3
SAMN32868503	SRR23185835	WS1525H	ICMB00555	panicle emergence	28	water bath	1
SAMN32868495	SRR23185786	WS2525H	ICMB00555	panicle emergence	28	water bath	2
SAMN32868487	SRR23185795	WS3525H	ICMB00555	panicle emergence	28	water bath	3
SAMN32868501	SRR23185837	WS1542H	ICMB00555	panicle emergence	42	water bath	1
SAMN32868493	SRR23185788	WS2542H	ICMB00555	panicle emergence	42	water bath	2
SAMN32868485	SRR23185797	WS3542H	ICMB00555	panicle emergence	42	water bath	3

**Table 2:** Data deposited in figshare.

File	Description
ICMB00333_ICMB00555_fertility.PNG	Seed setting rates in ICMB00333 and ICMB00555 panicles that underwent control and high-temperature treatments
count_all_PRJNA926343.txt	Read counts for all the genes and samples used for the analysis
tpm_all_PRJNA926343.txt	TPM for all the genes and samples used for the analysis
DEGs_ICMB00333_ICMB00555.zip	A file containing multiple compressed files, each of which shows a list of the DEGs obtained from a comparison between control and high temperature-treated samples

## 4 Experimental Design, Materials and Methods

### 4.1 Plant materials and high temperature treatments

Seeds of ICMB00333 and ICMB00555 were sown on a mixture of equal volumes of soil and vermiculite in perforated pots. Plants were grown in a growth chamber under the 12-hour 28°C day/12-hour 20°C night condition until they reached the booting, panicle emergence or anther emergence stage. For the 42°C treatment in a growth chamber, the plants in a control group were further grown for 48 hours under the same condition, and the other plants were grown for 48 hours under 12-hour 42°C day/12-hour 30°C night condition. For the 42°C treatment in a water bath, panicles of the plants at one of the above developmental stages were incubated in either 28°C or 42°C water in a water bath for 30 seconds. Florets in the middle part of the panicles of these plants were sampled immediately after the above treatments were finished, and stored at -80°C until they were used for RNA extraction.

### 4.2 RNA extraction, sequencing and data analyses

The florets sampled were ground in liquid nitrogen with a mortar and pestle to fine powders. Total RNA was extracted from them with the NucleoSpin RNA Plant kit (Macherey-Nagel, Düren, Germany). The resulting RNA samples were sent to Novogen Co. (Beijing, China) for mRNA sequencing by Illumina NovaSeq 6000 to obtain 150-base-paired-end reads for approximately 5Gb/sample. The resulting clean reads were mapped to the pearl millet reference genome [3] by Bowtie 2 with the –very-sensitive” option [4]. Read counts for each gene were then obtained by featureCounts [5]. TPM were obtained from these read counts by a custom Perl script. Genes with TPM more than two times greater or smaller under the 42°C condition than under the 28°C condition were extracted as DEGs. Scripts used for these analyses can be provided upon request.

## Limitations

None.

## Ethics Statement

This work meets the ethical requirements for publication in Data in Brief. This work does not involve human subjects, animal experiments, or any data collected from social media platforms.

## CRediT authorship contribution statement

**Xichao Lou:** Investigation, Data curation, Visualization, Writing – original draft. **Shashi Kumar Gupta:** Investigation, Data curation, Writing – original draft. **Tetsuo Takano:** Supervision, Conceptualization, Writing – review & editing. **Daisuke Tsugama:** Investigation, Data curation, Supervision, Conceptualization, Writing – original draft.

## Data Availability

RNA sequencing of florets of the pearl millet high temperature-tolerant line ICMB00333 and the high temperature-sensitive line ICMB00555 (Original data) (NCBI BioProject) Supplementary data for RNA sequencing with two pearl millet cultivars with different tolerance to a high temperature (Original data) (figshare)
